# Deep brain stimulation for substance use disorder: a systematic review and meta-analysis

**DOI:** 10.3389/fpsyt.2023.1231760

**Published:** 2023-08-10

**Authors:** Nour Shaheen, Ahmed Shaheen, Can Sarica, Arun Singh, Mario Zanaty, Karim Johari, Andrew Yang, Theresa Zesiewicz, Brian Dalm, Yarema Bezchlibnyk, Andres M. Lozano, Oliver Flouty

**Affiliations:** ^1^Alexandria Faculty of Medicine, Alexandria, Egypt; ^2^Division of Neurosurgery, University of Toronto, Toronto, ON, Canada; ^3^Division of Basic Biomedical Sciences, Sanford School of Medicine, University of South Dakota, Vermillion, SD, United States; ^4^Department of Neurological Surgery, Thomas Jefferson University Hospitals, Philadelphia, PA, United States; ^5^Department of Communication Sciences and Disorders, Louisiana State University, Baton Rouge, LA, United States; ^6^Department of Neurosurgery and Brain Repair, University of South Florida, Tampa, FL, United States; ^7^Department of Neurological Surgery, Ohio State University, Columbus, OH, United States

**Keywords:** addiction, deep brain stimulation, substance abuse, alcohol, heroin, tobacco

## Abstract

**Objective:**

Substance use disorder (SUD) is a significant public health issue with a high mortality rate. Deep brain stimulation (DBS) has shown promising results in treating SUD in certain cases. In this study, we conducted a meta-analysis to evaluate the efficacy of DBS in the treatment of SUD and reduction of relapse rates.

**Methods:**

We performed a thorough and methodical search of the existing scientific literature, adhering to the PRISMA guidelines, to identify 16 original studies that fulfilled our inclusion criteria. We used the evidence levels recommended by the Oxford Centre for Evidence-Based Medicine to assess bias. The R version 4.2.3 software was utilized to calculate the mean effect size. We estimated study heterogeneity by employing tau2 and I2 indices and conducting Cochran’s Q test.

**Results:**

The results showed that DBS treatment resulted in a significant improvement in the clinical SUD scales of patients, with an average improvement of 59.6%. The observed relapse rate was 8%. The meta-analysis estimated a mean effect size of 55.9 [40.4; 71.4]. Heterogeneity analysis showed a large degree of heterogeneity among the included studies. Subgroup and meta-regression analysis based on age and SUD type suggested that DBS may be more effective for patients above 45 years of age, and for alcohol and opioid addiction compared to nicotine addiction.

**Conclusion:**

The current literature suggests that DBS has a moderate effect on SUD symptoms. However, the limited number of studies and small sample size indicate that more research is needed to better understand the factors that influence its effectiveness.

## Introduction

Substance use disorder (SUD) is a chronic medical condition characterized by persistent substance abuse despite adverse consequences. This condition poses a substantial global health concern, as underscored by the Global Burden of Disease (GBD) study in 2015 (1). Tobacco smoking has the highest mortality rate (110.7 deaths per 100,000 people), followed by alcohol and illicit drugs (33.0 and 6.9 deaths per 100,000 people, respectively) (2, 3). The impact of SUD is not limited to health outcomes but also poses significant economic consequences. For example, the opioid epidemic in the United States alone resulted in an economic burden of $1.02 trillion in 2017 (4).

Despite the existence of multiple approaches for treating SUD, their effectiveness has been suboptimal. Achieving long-term abstinence from substance use through medical interventions has been a difficult task, with pharmacological treatments showing limited success in reducing drug use or criminal behaviors. Studies have shown that approximately 85% of individuals who struggle with addiction experience a relapse within the first year after attempting abstinence.

During the 1960s, neurosurgical procedures such as cingulotomy, hypothalamotomy, and substantia innominate resection were attempted as treatments for SUD; however, their success rates were inconsistent (5). Nevertheless, these procedures fell out of favor due to the introduction of new pharmacological treatments and ethical concerns surrounding their use (6, 7). Advancements in DBS methodology and safety have raised interest in its use for treating neuropsychiatric disorders, including SUD, depression (8), obsessive–compulsive disorder (OCD) (9), and Tourette syndrome (10). DBS shows promise as a treatment for SUD, particularly for those with severe and persistent opioid, benzodiazepine, alcohol, cocaine, heroin and nicotine addiction. Existing evidence on DBS for SUD mostly relies on small cohort studies, making it challenging to draw clear conclusions.

Our study aims to review and meta-analyze the SUD literature to better understand the effectiveness of DBS and its influencers on SUD in its most severe form: addiction. Addiction, specifically, refers to a complex brain disorder that is a subset of substance use disorders. It is characterized by compulsive drug-seeking behavior, continued substance use despite negative consequences, and a loss of control over substance use. In other words, addiction is a chronic and relapsing condition that involves a compulsive drive to seek and use a substance, even when it becomes detrimental to an individual’s physical and mental health, relationships, and overall well-being.

## Methods

This systematic review and meta-analysis followed the Cochrane Handbook for Systematic Reviews of Interventions (11) and adhered to the Preferred Reporting Items for Systematic Reviews and Meta-Analyses (PRISMA) guidelines, ensuring a rigorous and standardized approach to the study’s methodology (12).

### Systematic literature search

Two authors (OF and NS) conducted an independent systematic search of various databases including PubMed, Scopus, OVID, and Web of Science using a comprehensive search strategy to identify studies that investigated the effect of DBS on addiction treatment. The search was conducted using medical subject headings (MeSH) terms and keywords related to “addiction” and “Deep brain stimulation,” covering the years from 1950 to 3 April 2023. The search strategy included terms such as “addiction” and “DBS” in various combinations and was applied to different databases (Supplementary material S1).

The analyzed studies specifically investigated the effects of deep brain stimulation (DBS) on the treatment of addiction to alcohol, smoking, and drugs. The effect of DBS on alcohol use disorder treatment was evaluated by comparing mean difference scores before and after a minimum of 12 months of DBS. Additional requirements for inclusion in the analysis were that the articles had to be written in English, published in a peer-reviewed scholarly journal, and focused on studies involving interventions or treatments, rehabilitation, or epidemiological examinations. Articles based on animal studies, reviews, descriptive articles, book chapters, or technical notes were identified and excluded individually from the meta-analysis.

### Data synthesis and statistical analysis

The meta-analysis evaluated the included studies and systematically examined their outcome measures. The quality assessment of the studies was conducted using the Oxford Centre for Evidence-Based Medicine levels of evidence (levels I–V) (13). Since the studies used different outcome measures, the reported means and variances of the treatment before and after surgery were used to calculate treatment effect sizes (Cohen’s d) and the standard error of the effect sizes (14). Some studies reported multiple outcome measures, including cognitive testing, clinical response, and psychometric response for addiction treatment improvement.

To combine the results, the effect size and standard deviation were calculated separately for each outcome measure and then averaged to provide a single outcome value per study due to the heterogeneity of the measures. The heterogeneity between studies was tested using Cochran’s Q test, and study heterogeneity was estimated using tau^2^ and I^2^ indices. We used R version 4.2.3 to calculate the mean effect size using the metamean() function. Random-effects models were used to account for heterogeneity among studies. Additionally, subgroup or meta-regression analyses were conducted based on the ‘Drug’, ‘Years of addiction’ and ‘Age’ variables using the ‘byvar’ argument. The results were presented as forest plots, and a funnel plot was used to display publication bias.

## Results

### Systematic search and summary of findings

**Figure 1:**
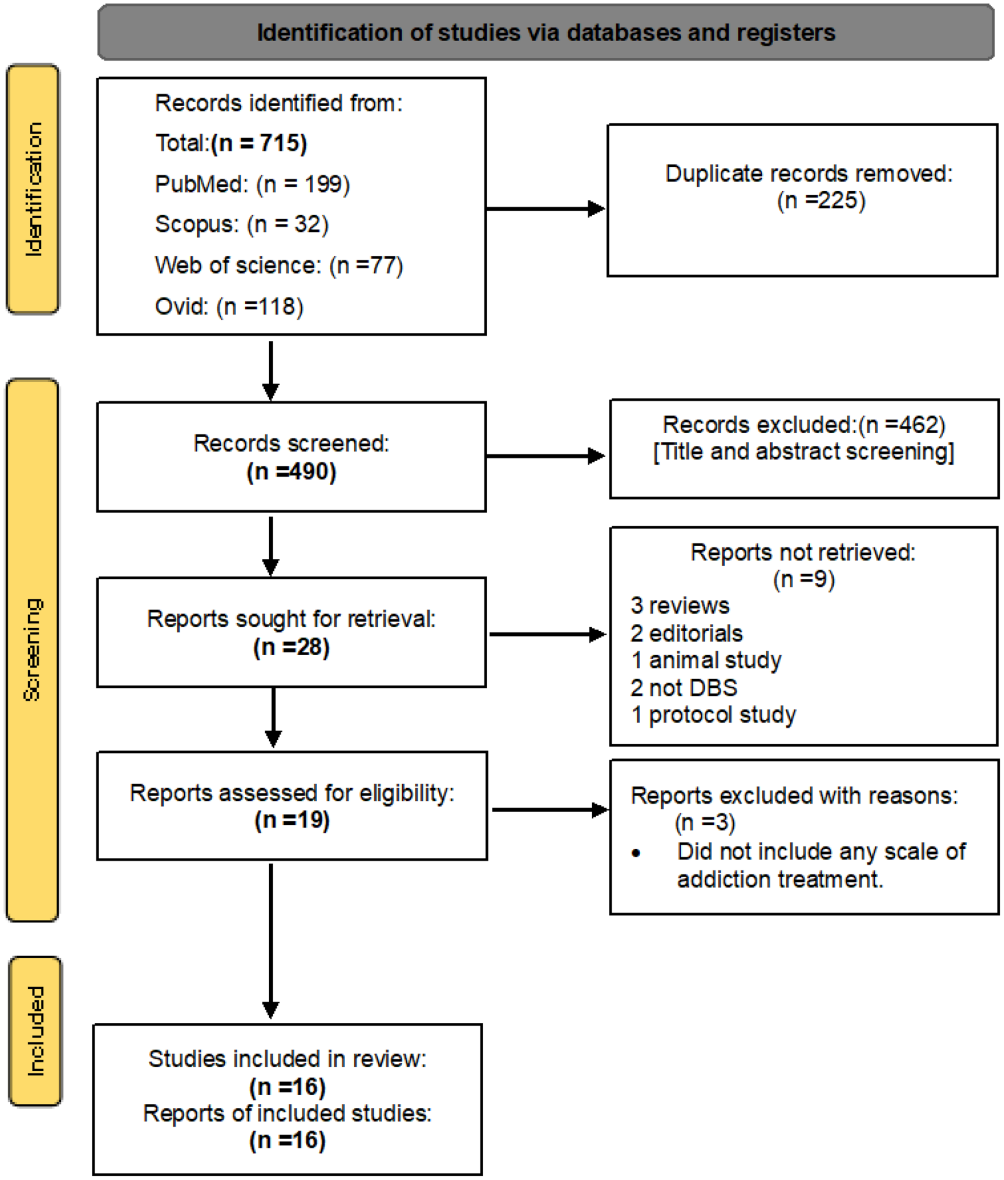
PRISMA 2020 flow diagram for new systematic reviews which included searches of databases and registers only.

Searching databases resulted in a total of 199 records from PubMed, 321 records from Scopus, 77 records from Web of Science Core Collection, and 118 records from OVID, including related terms. After eliminating any duplicated articles, a selection of 490 journal articles were chosen based on their relevance to DBS as a form of addiction treatment. These were then further screened according to our specific inclusion criteria, resulting in 16 original research studies that were deemed potentially eligible for inclusion in our study (Figure 1). These 16 studies were ultimately included in our final analysis, as outlined in Table 1.

**Table 1:** Characteristics of studies on DBS for addiction treatment, including the author, year, study design, number of participants, sex ratio, mean age, drug used, dosage, years of addiction, response to detoxification, target of DBS, follow-up duration, relapse rate, lateralization, and percentage of improvement in addiction symptoms.

Study ID	Study design	No.	Sex-male (M)	Age	Drug	Dose	Years of addiction	Response to detoxification	Target	FU	Relapse	Outcome measurement	Improvement (%)	
													Mean	SD
Bach et al. 2023 (15)	Double blinded RCT	5	5 M	44.2 (9.9)	Alcohol	15.6 (12.1) per day	10	No	NAc	18 months	0	HAMD, HAMA, FTND, BDI-II, SHAPS, Physical Anhedonia Social Anhedonia, WHOQOL-BREF, GAF, OCDS, AUDIT, ADS, AUQ	47.9	28.2
Davidson et al. 2022 (16)	prospective cohort	6	4 M	49 + −12.1	Alcohol	9/day	16.3	No	NAc	12 months	2	BAI, HAMD, OCDS, TLFB, AUDIT, ACQ, ADS	49.5	20.9
Mahoney et al. 2021 (17)	Clinical Trial	1	1 M	30	Opioid and benzodiazepine		10	No	NAc/VC	12 months	0	HRV (rMSSD), BART, BIS-11, MADRS, BSA, VAS	95.02	55.5
Zhu et al. 2020 (18)	Case report	1	M	28	Drugs [bucinnazine/morphine]	1,200 mg per day/200 mg morphine every 2 months	13	No	NAc	12 months	0	HAMD, HAMA, BAI	61.4	7.3
Chen et al. 2019 (19)	Clinical Trial	8	7 M	34 (8.8)	Heroin	0.21 (0.06) per day	12.6 (7.4)	No	ALIC into the NAc	24 months	2	VAS, HDRS-17, Y-BOCS, SF-36, SCL-90	62.5	26.7
Müller et al. 2016 (20)	Clinical Trial	3	2 M	37.3	Alcohol		20.4	No	NAc	6 months	0	ADS, AUQ	44.2	41.9
Gonçalves-Ferreira et al. 2016 (21)	RCT [editorial]	1	M	36	Cocaine		16		NAc	30 months	0	VAS, Y-BOCS, DDQ Desire and Intention to Use Cocaine, DDQ Negative Reinforcement of Cocaine Use, CGI	90.2	13.6
Kuhn et al. 2014 (22)	Case report	2	1 M	32	Opioid			No	NAc	2 years	0	HAMA, BDI-II, VAS	54.4	30.9
Voges et al. 2013 (23)	Case report	5	5 M	44	Alcohol		26.2	No	NAc	38 months	0	OCDS, ADS, GSI of the SCL-90 Score, AUQ	64.6	50.04
Valencia-Alfonso et al. 2012 (24)	Case report	1	1 M	47	Heroin	0.5 g/day	22	No	NAc	6 months	0	DI Score	30.3	0
Kuhn et al. 2011 (25)	Case report	1	M	69	Alcohol	200 g of vodka per day	30	No	NAc	12 months	0	OCDS, CDT, CBQ, DITS, OCI-R, WST (~IQ), AUDIT, ADS	56.6	33.09
Zhou et al. 2011 (26)	Case report	1	1 M	24	Heroin	1.0 g to ∼1.5 g per day	5	No	NAc	3 months	0	WMS, full IQ, verbal IQ, performance IQ	11.49	3.34
Mantione et al. 2010 (27)	Case report	1	1\u00B0F	47	Nicotine				NAc	2 years	0	HAMD, HAMA, Y-BOCS	84.14	12.4
Kuhn et al. 2009 (28)	Clinical Trial	10	7 M	43.5 (SD 8.48)	Nicotine		10	No	NAc	13.5 (SD = 14.85)	0	FTND, Abstinence Motivation	14.9	14.2
Müller et al. 2009 (29)	Case report	3	3 M	37.6	Alcohol	2 L of hard liquor a day, 10–15 L of beer a day	25.6	No	NAc	12 months	0	OCDS, ADS, AUQ	90	14.14
Kuhn et al. 2007 (30)	Case report	1	1 M	54	Alcohol	10 drinks per day	10	No	NAc	13 months	0	BDI-II, AUDIT	96.42	0

HAMD, Hamilton Rating Scale for Depression; HAMA, Hamilton Rating Scale for Anxiety; FTND, Fagerström Test for Nicotine Dependence; BDI-II, Beck Depression Inventory - Second Edition; SHAPS, Snaith-Hamilton Pleasure Scale; Physical Anhedonia, Scale for physical anhedonia assessment; Social Anhedonia, scale for social anhedonia assessment; WHOQOL-BREF, World Health Organization Quality of Life – BREF; GAF, Global Assessment of Functioning; OCDS, Obsessive–Compulsive Drinking Scale; AUDIT, Alcohol Use Disorders Identification Test; ADS, Alcohol Dependence Scale; AUQ, Alcohol Use Questionnaire; BAI, Beck Anxiety Inventory; TLFB, Timeline Follow-Back; ACQ, Anxiety Sensitivity Index; HRV (rMSSD), Heart Rate Variability (root mean square of successive differences); BART, Balloon Analog Risk Task; BIS-11, Barratt Impulsiveness Scale - Version 11; MADRS, Montgomery-Åsberg Depression Rating Scale; BSA, Body Surface Area; VAS, Visual Analog Scale; HDRS-17, Hamilton Depression Rating Scale - 17 items; Y-BOCS, Yale-Brown Obsessive Compulsive Scale; SF-36, Short Form Health Survey - 36 Items; SCL-90, Symptom Checklist-90; DDQ, Desire and Intention to Use Cocaine; CGI, Clinical Global Impression; GSI, Global Severity Index of the SCL-90 Score; DI Score, Dependency Inventory Score; CDT, Clock Drawing Test; CBQ, Cocaine Craving Questionnaire; DITS, Delayed-Imitation Test of Speech; OCI-R, Obsessive–Compulsive Inventory – Revised; WST (~IQ), Wechsler Short Test for Intelligence (~IQ estimation); WMS, Wechsler Memory Scale; IQ, Intelligence Quotient; VAS, Visual Analog Scale; Abstinence Motivation, scale for assessing motivation for abstinence.

The study analyzed data from 16 independent studies, comprising a total of 50 patients. The mean age of the patients was 43.2 years (standard deviation [SD] = 13.7). Forty-one were male, and the mean duration of follow-up was 16.2 months (SD = 9.4). On average, the patients had a history of addiction lasting 16.6 years (SD = 7.38). The patients exhibited a significant improvement in their clinical addiction scales following deep brain stimulation (DBS) treatment, with an average improvement of (59.6 ± 26.8) %. The abstinence rate observed among the patients was 92% (Table 1).

### Pooling analysis

**Figure 2:**
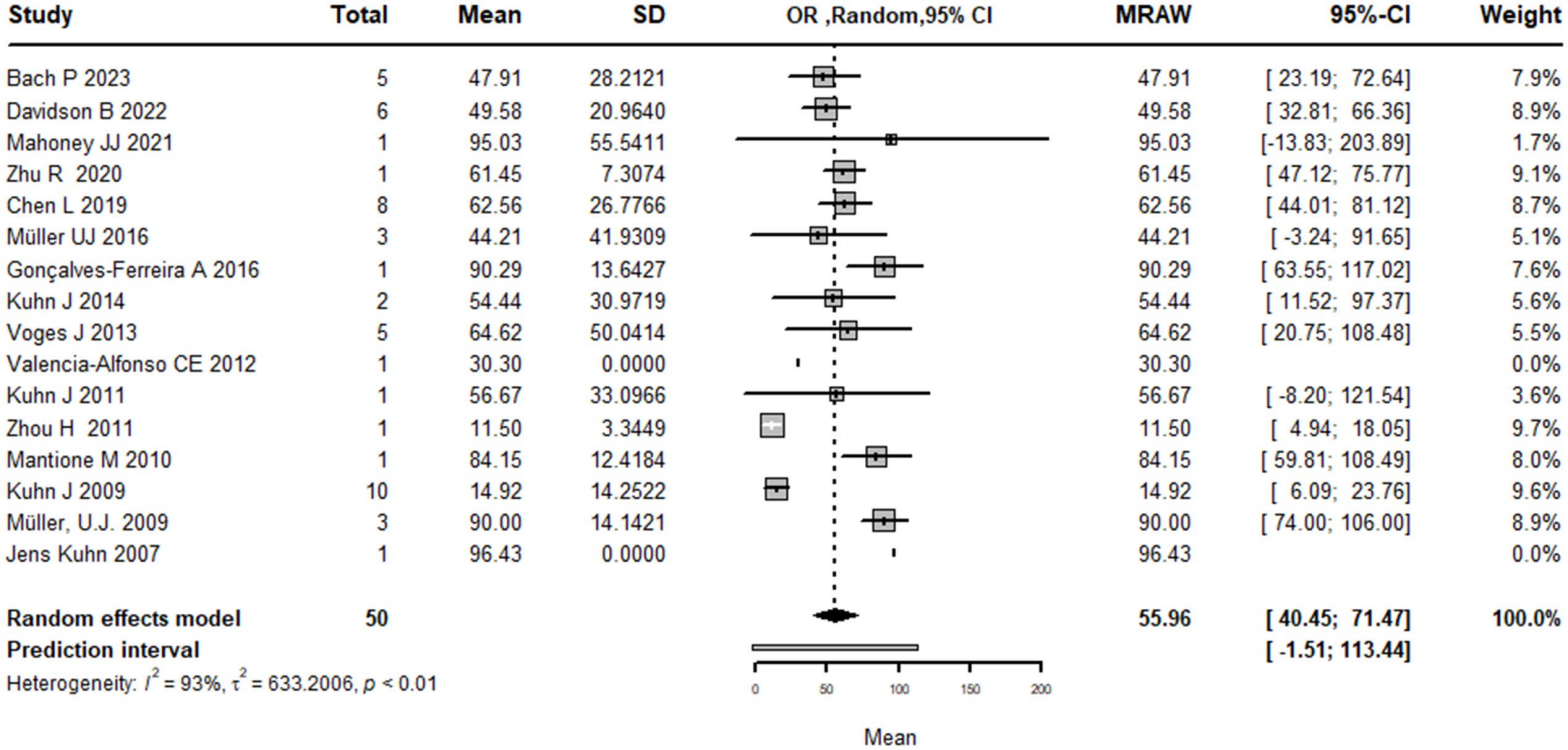
Forest plot graph of the pooled analysis of all included studies showing the mean improvement of the addiction clinical scales after DBS (Deep Brain Stimulation).

**Figure 3:**
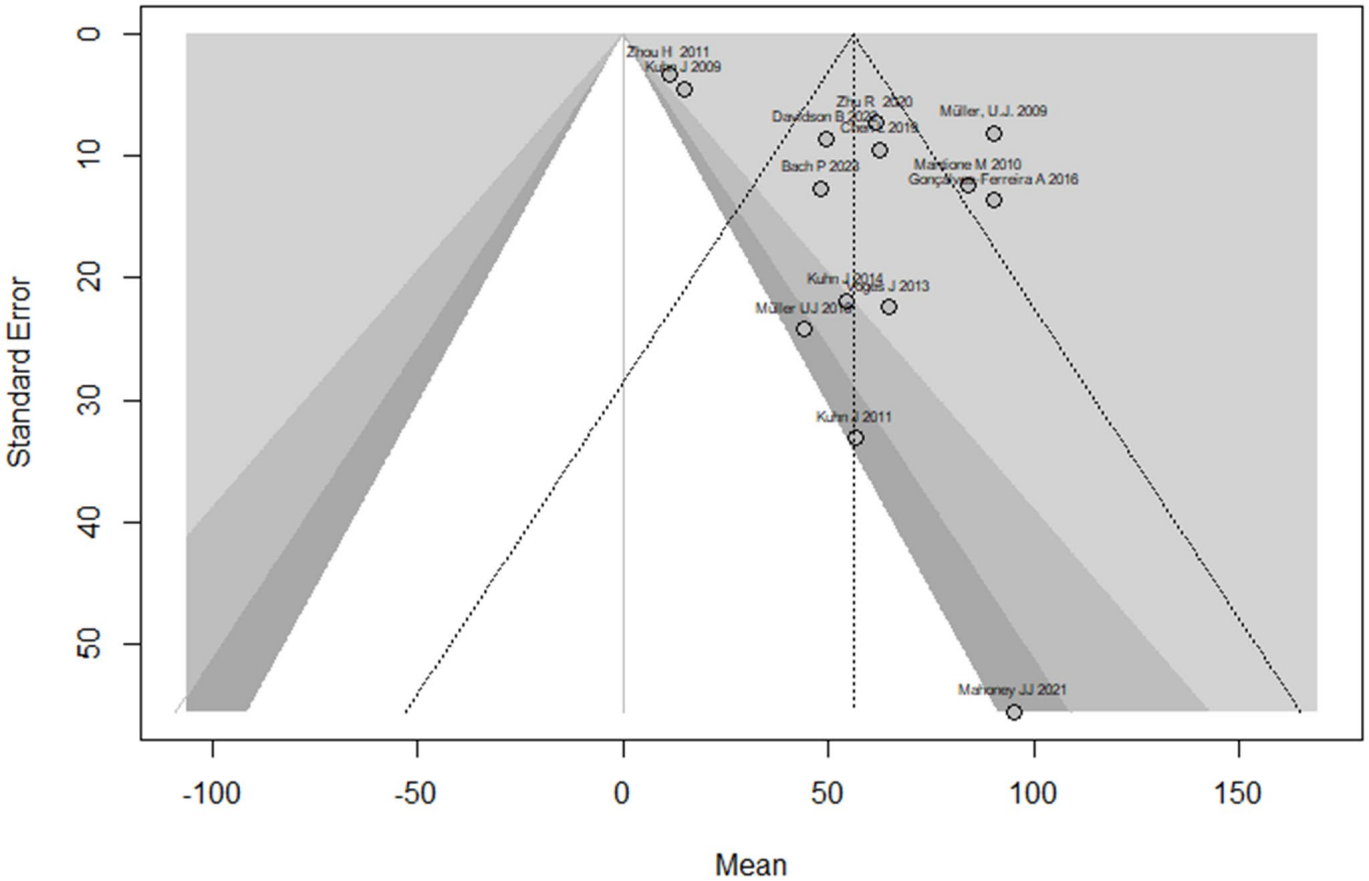
The funnel plot displays the treatment effect estimations in comparison to the standard error of the impact size of each study.

This systematic review and meta-analysis included 16 studies with a total of 50 observations on the use of deep brain stimulation (DBS) for addiction treatment. The analysis used a random effects model, which estimated a mean effect size of 55.9 with a 95% confidence interval of [40.4; 71.4]. The results suggest that DBS is effective for addiction treatment. The analysis also quantified the heterogeneity of the studies, which refers to the variability in effect sizes across studies. The tau^2^ statistic, which estimates the between-study variance, was 633.2 with a 95% confidence interval of [231.4; 1456.4]. The tau statistic, which is the square root of tau^2^ and represents the standard deviation of the true effects across studies, was 25.1 with a 95% confidence interval of [15.2; 38.1]. The *I*^2^ statistic, which represents the proportion of total variability due to heterogeneity, was 92.6% with a 95% confidence interval of [89.3%; 94.9%]. The H statistic, which is the ratio of the total variance to the within-study variance, was 3.7 with a 95% confidence interval of [3.1; 4.4]. These statistics indicate substantial heterogeneity among the studies. The analysis tested the heterogeneity using the Q statistic, which was175.7 with 13 degrees of freedom and a value of *p* < 0.0001, indicating significant heterogeneity (Figure 2). The linear regression test of funnel plot asymmetry yielded a significant result (*t* = 3.0, *df* = 12, value of *p* = 0.01), indicating the presence of publication bias. The sample estimates for the bias and intercept were 3.9 (se = 1.3) and 8.3 (se = 10.2), respectively. The test was conducted using a multiplicative residual heterogeneity variance (tau^2 = 8.3) and the predictor was the standard error, with weights based on the inverse variance. The reference for this test was Egger et al. (31), published in BMJ (Figure 3).

### Subgrouping analysis

#### Age meta-regression analysis

The analysis also examined the effect of age as on the treatment effect of DBS on addiction by conducting subgroup analyses based on different age groups. The results showed that the mean effect size varied widely across age groups, ranging from 11.4 [4.9; 18.1] for the youngest age group (24 years) to 95.0 [−13.8; 203.8] for the second youngest age group (30 years). However, the confidence intervals for some of the subgroups were wide and did not reach significance, indicating that the true effect size of age should be interpreted with caution and remains uncertain.

**Figure 4:**
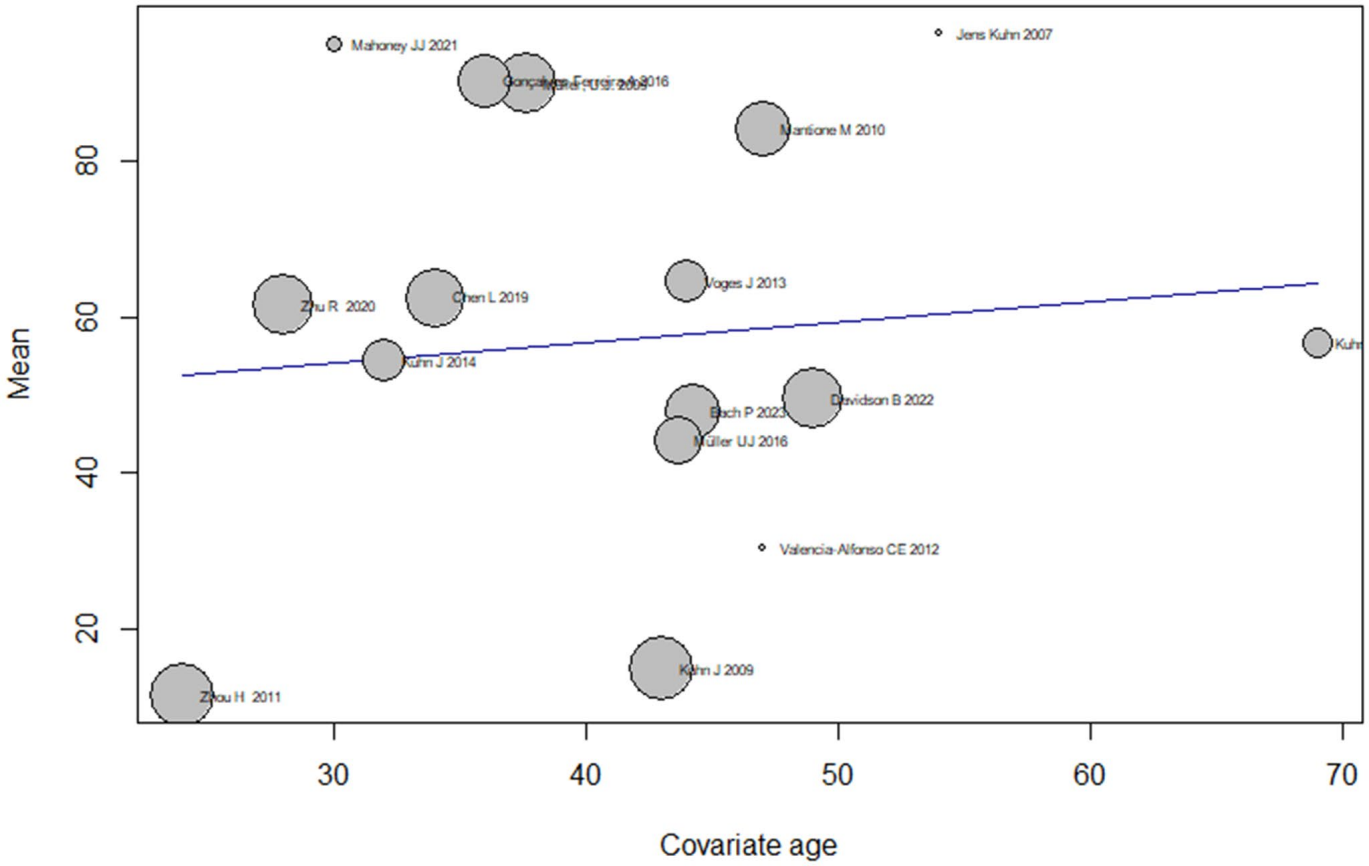
Age to DBS addiction treatment meta-regression analysis.

The test for subgroup differences indicated that there was significant heterogeneity in effect sizes across the age subgroups (*Q* = 175.86, *df* = 13, *p* < 0.0001), suggesting that age may be an important moderator of the treatment effect of DBS on addiction. Overall, the meta-regression analysis suggests that DBS may have a moderate effect on addiction symptoms, but the treatment effect may vary depending on age (Figure 4).

#### Subgrouping by substance type

Using types of substance use disorder as the basis for subgroup analysis, the results show that the mean effect size of DBS for addiction treatment was higher for alcohol use disorder 61.3 [42.4; 80.1] and opioid use disorder 56.4 [30.4; 82.3] compared to nicotine use disorder 48.5 [−19.2; 116.3]. However, it is important to note that the confidence intervals for nicotine use disorder are wide and cross zero, indicating that the true effect size is uncertain.

The analysis also shows that there is significant heterogeneity among the studies, with a large tau^2^ value (indicating substantial variability in effect sizes) and a high I^2^ value (indicating a high degree of heterogeneity). The subgroup analysis suggests that the heterogeneity may be partially explained by differences in the type of drug addiction being treated, with greater heterogeneity observed for nicotine use disorder compared to alcohol and opioid use disorder.

The test for subgroup differences did not find a significant difference between the substance use disorder types, indicating that the effect of DBS on addiction treatment did not differ significantly between alcohol, opioid, and nicotine use disorders.

**Figure 5:**
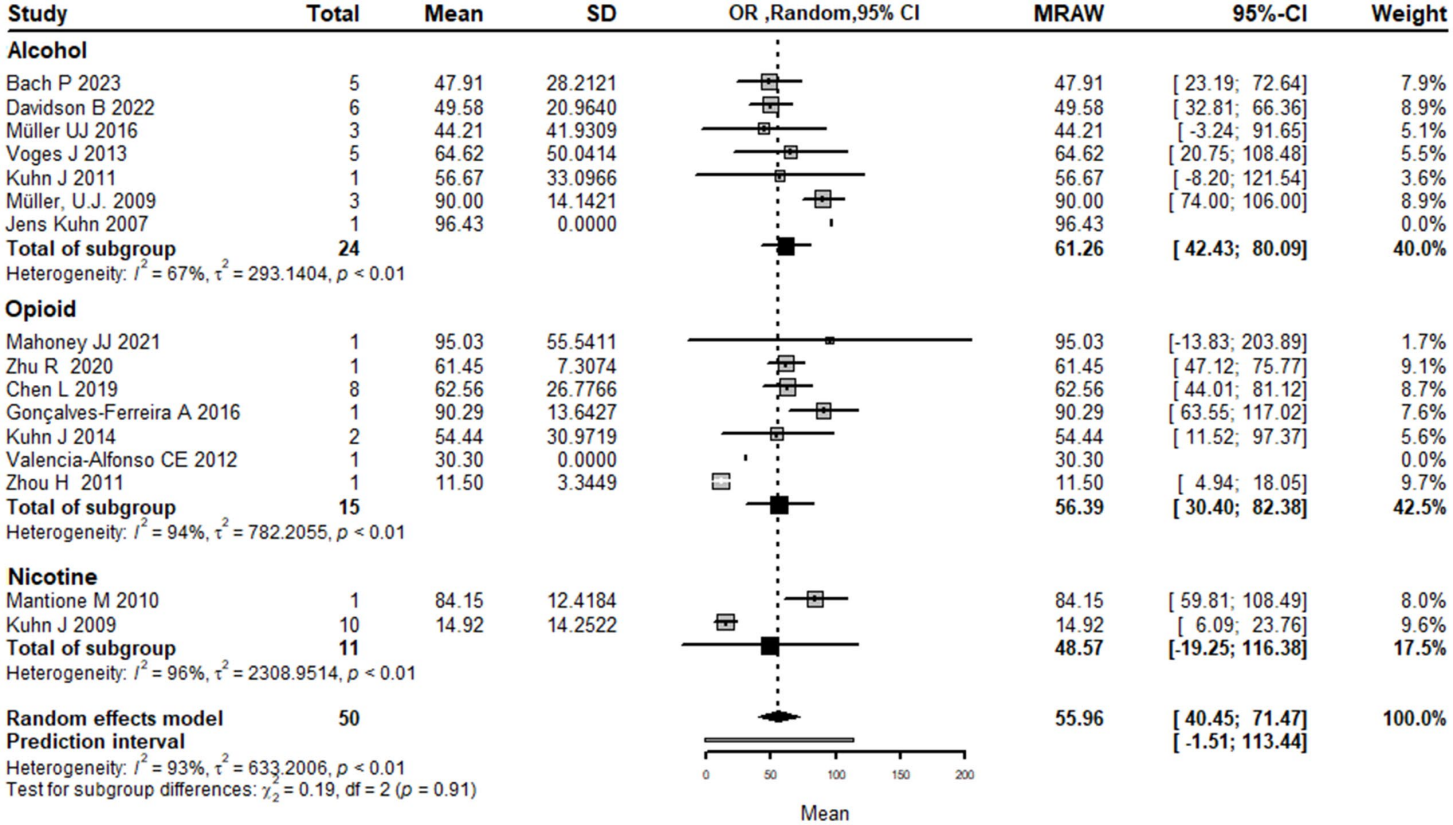
Forest plot graph of the pooled analysis of all included studies with Drugs addicted subgrouping showing the mean improvement of the addiction clinical scales after DBS.

Overall, while the results suggest that DBS may be more effective for alcohol and opioid use disorder compared to nicotine use disorder, the high degree of heterogeneity and the limited number of studies in the subgroup analysis make it difficult to draw definitive conclusions from the current literature (Figure 5).

#### Duration meta-regression analysis

Subgroup analyses based on different durations of addiction show that the mean effect size of DBS on addiction treatment varies across subgroups. However, most subgroups have a wide confidence interval that overlaps with the overall effect size estimate. The subgroup with 16.0 years of addiction has the highest mean effect size of 49.5 [32.8; 66.3], while the subgroup with 5.0 years of addiction has the lowest mean effect size of11.5 [4.9; 18.1].

The test for subgroup differences suggests that there is significant heterogeneity across subgroups (*Q* = 152.6, *p* < 0.0001), indicating that the duration of addiction is a significant moderator of the effect of DBS on addiction treatment.

**Figure 6:**
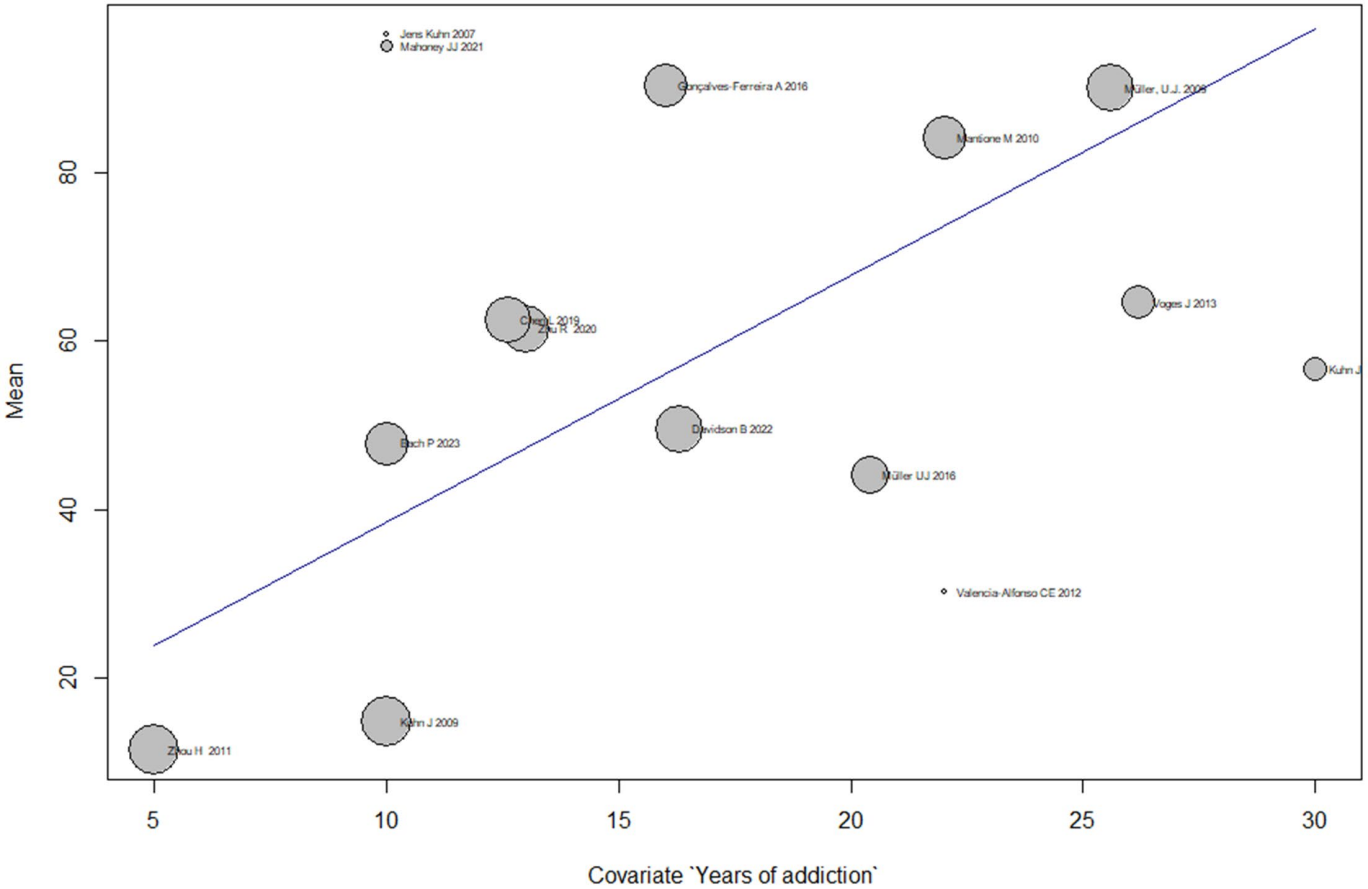
Duration to DBS addiction treatment meta-regression analysis.

Overall, the meta-regression suggests that the effect of treating addiction by DBS varies based on the duration of addiction, and the duration of addiction is an important moderator of the effect size of DBS on addiction treatment (Figure 6).

### Sensitivity analysis

**Figure 7:**
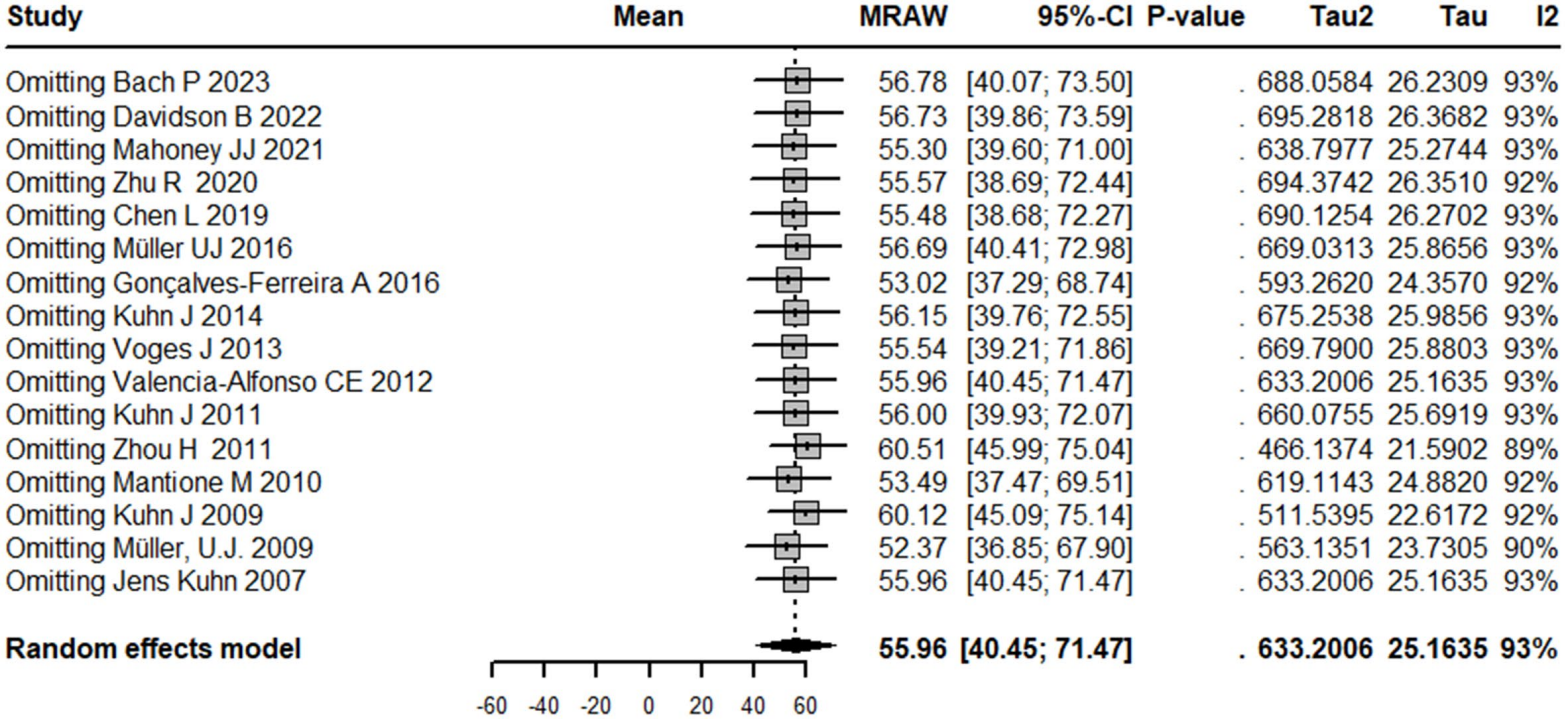
Sensitivity analysis graph showing the results of overall mean estimate in case omitting any study separately.

The results of the sensitivity analysis with the random effects model indicate that the pooled estimate of the meta-analysis is 55.9 [40.4; 71.4]. The influential analysis shows the estimated mean and 95% CI of the pooled estimate when one study is omitted at a time. The results suggest that the pooled estimate is sensitive to the exclusion of any of the studies, with the largest change observed when omitting Zhou H 2011, which leads to a pooled estimate of 60.5 [46.0; 75.0]. The random effects model also estimates the amount of between-study heterogeneity with tau^2^ and I^2^. The results indicate that the pooled estimate has a high degree of between-study heterogeneity, with tau^2^ estimated at 25.2 and *I*^2^ estimated at 92.6% (Figure 7).

## Discussion

The concept that targeted brain lesioning or natural brain injuries could lead to the elimination of addictive behavior is not a recent idea. Joutsa et al. conducted a study that explored how brain damage in specific regions can induce remission from drug addiction. The study suggests that investigating these cases can aid in the identification of new treatment targets. By analyzing data from two patient groups with smoking addiction and brain damage, researchers identified a disrupted brain circuit that was linked with addiction remission. This circuit was characterized by positive connectivity to certain regions and negative connectivity to others, and its hubs could potentially be targeted for therapeutic neuromodulation. The results indicate that brain lesions causing addiction disruption map to a specific circuit in the human brain, providing potential targets for novel treatments (32). Addiction is characterized by a persistent desire to consume a substance or engage in a behavior, known as craving, which can be difficult to quantify through self-reported measures alone. However, functional magnetic resonance imaging (fMRI) has enabled researchers to identify a neurobiological marker, referred to as the Neurobiological Craving Signature (NCS), which is associated with cue-induced cravings for drugs and food. The NCS is based on activity patterns in distinct brain regions and may hold promise as a tool for developing clinical interventions aimed at reducing cravings and preventing relapse (33).

Individuals who are chronically dependent on stimulants, as well as their nondependent siblings, have demonstrated a decrease in the size of their insula (34). The patients in these instances did not need to exert any self-control to resist the urge to smoke since they did not experience any desire to smoke. This similarity between the suppression of cravings resulting from anterior cingulate and NAc surgery may not be accidental as both the insula and the anterior cingulate are closely linked to the NAc (35). At the cellular level, addictive drugs alter the levels of dopamine outside the synapses within the “DA motive system,” with the NAc being the primary area affected (36). Continued usage of these substances causes changes at the cellular level, such as in the anterior cingulate and orbitofrontal cortices (37). These changes contribute to the desire to seek out drugs and the likelihood of relapse, which are defining features of drug addiction (37). The NAc has been separated both anatomically and functionally into two distinct parts: the core, which is situated in the ventral and medial area, and the shell, which is found in the lateral region of the NAc (38). The NAc shell area obtains inputs from the limbic system that originate from the basolateral amygdala (BLA) and ventral subiculum. It primarily transmits these inputs to the preoptic area of the hypothalamus. On the other hand, the NAc core region receives inputs from the BLA and parahippocampal cortex and sends outputs to motor circuits (39). According to the present evidence, it is believed that the shell regulates reward or drug-seeking behavior by utilizing spatial/contextual information, while the core regulates these behaviors through specific signals (40). Furthermore, *in vivo* microdialysis experiments on animals suggest that drug abuse has varying effects on the core and shell areas. The shell shows a significant increase in dopamine concentrations, while the core experiences only a slight increase (41). Research indicates that dopamine neurotransmission plays a vital role in reinforcing drug use (42).

Traditionally, the most popular DBS target for treating addiction is the NAc. Electrodes implanted in this region deliver electrical impulses that are thought to modulate the brain activity and reduce substance-related cravings and compulsions (43). Luigjes and co-authors conducted a review of potential DBS targets for substance use disorder and identified six brain regions that were studied in seven animal studies and two brain regions in eleven human studies. These regions included the NAc, STN, dorsal striatum, lateral habenular nucleus, mPFC, and hypothalamus. Based on their analysis, they determined that the NAc is the most promising target for DBS treatment in patients with addiction that has been unresponsive to other therapies (44). According to our meta-analysis, all patients received DBS targeting the NAc, except for Chen (19) who reported using stimulation in the anterior limb of the internal capsule (ALIC) in addition to the NAc. The NAc is a commonly targeted area for the treatment of OCD and depression as well, suggesting a shared involvement of the NAc in various psychiatric disorders involved in reward processing (45). However, the way in which NAc DBS treatment works for addiction is not yet fully understood. According to several animal studies, it appears that the decrease in addictive behavior relies on the restoration of normal activity in the NAc shell, which is the region of the NAc responsible for processing the pleasurable effects of drugs (46, 47). However, the mechanism behind the treatment of addiction through NAc DBS remains uncertain. Additionally, research has revealed that the impact of NAc shell DBS is reliant on modifying the activity in the prefrontal cortex, which is located upstream (46–49). A recent study showed that DBS of the NAc shell resulted in a minor increase in cocaine self-administration and a reduction in irritability-like behavior during cocaine withdrawal. This finding suggests that DBS may not reduce cocaine intake in individuals with long-term cocaine use but may be beneficial in managing negative emotional symptoms that arise during cocaine abstinence (50).

To our knowledge, this is the first and largest meta-analysis that investigated the impacts of DBS for the treatment of addiction, which included 16 studies and 50 subjects. The analysis estimated a mean effect size of 56.0 with a 95% confidence interval of [40.5; 71.5], indicating that DBS is effective for addiction treatment, but there is substantial heterogeneity among the studies. The study also performed subgroup analyses based on age, type of substance use, and duration of addiction, and the results showed significant differences in the mean effect size for different subgroups. The sensitivity analysis indicated that the pooled estimate was not strongly influenced by any individual study.

The results of the pooling analysis are important clinically and for research because they suggest that DBS could serve as an effective treatment for addiction, which is a chronic and relapsing brain disease that affects millions of people worldwide. The findings have significance for healthcare providers and individuals seeking treatment since they indicate that DBS could be a potential strategy for addressing addiction, particularly in cases where patients are treatment resistant. The use of DBS in the treatment of addiction was associated with a relapse rate of 8%, compared to the typical 85% relapse rate observed in early abstinence (51, 52). The meta-analysis discovered that the length of addiction plays a crucial role in determining the effectiveness of DBS in treating addiction, which is consistent with the principles of medical treatment that consider addiction duration and response to therapy. The duration of addiction can impact the probability of attaining and sustaining abstinence from drugs or alcohol. Scott et al. (53) have shown that even occasional abstinence from drugs and alcohol is linked to decreased mortality risk. Long-term treatment has a direct and indirect influence on mortality, with the duration of sustained abstinence being a significant factor (53).

The results of the pooling analysis show that DBS has a large and significant effect size on reducing drug craving and consumption, but also reveal substantial heterogeneity among the studies, indicating that there are differences in the outcomes depending on factors such as age, type of drug, and duration of addiction. These differences suggest that DBS may not work equally well for all patients with addiction, and that there may be optimal parameters for selecting candidates, choosing target regions, and adjusting stimulation settings. Therefore, more research is needed to identify the mechanisms of action, the safety and ethical issues, and the long-term effects of DBS for addiction.

There are several factors that should be taken into consideration regarding the limitations of this study. Initially, the study’s meta-analysis only includes a limited number of studies (16) and has a restricted sample size (50 patients). This could have resulted in an analysis that lacked sufficient power, particularly in subgroup analyses, reducing the overall applicability of the findings.

Additionally, there is considerable variability among the studies incorporated in the analysis, which may impede the ability to arrive at firm conclusions. Despite attempts to explore potential causes of heterogeneity using subgroup analyses, the outcomes must be viewed with caution, as the limited number of studies in each subgroup and the considerable level of variability noted may impact their reliability.

## Conclusion

We investigated the current literature and showed that DBS has favorable effects on addiction and may reduce clinical scores by up to 56%. Moreover, DBS seems to reduce relapse to 8% compared to the 85% quoted relapse rate observed in early abstinence. Nevertheless, the considerable heterogeneity among studies highlights the need for larger, standardized randomized controlled trials that explore the effect of DBS on addiction in greater detail, including the factors that contribute to these findings through subgroup analysis. Despite this limitation, this study indicates that DBS has the potential to serve as a valuable adjunct to best medical therapy or even as a standalone treatment for addiction.

## Author contributions

OF and NS contributed to conception and design of the study and wrote the first draft of the manuscript. NS organized the database and performed the statistical analysis. NS, OF, AL, AY, and AL wrote sections of the manuscript. All authors contributed to the article and approved the submitted version.

## Funding

This study was funded by the Department of Neurosurgery at the University of South Florida.

## Conflict of interest

AL is the scientific director of Functional Neuromodulation Ltd. and a consultant to Medtronic, Abbott, Boston Scientific, Insightec, and the Focused Ultrasound Foundation. CS has fellowship grants from Michael and Amira Dan Foundation and the Turkish Neurosurgical Society.

The remaining authors declare that the research was conducted in the absence of any commercial or financial relationships that could be construed as a potential conflict of interest.

## Publisher’s note

All claims expressed in this article are solely those of the authors and do not necessarily represent those of their affiliated organizations, or those of the publisher, the editors and the reviewers. Any product that may be evaluated in this article, or claim that may be made by its manufacturer, is not guaranteed or endorsed by the publisher.

## Supplementary material

The Supplementary material for this article can be found online at: https://www.frontiersin.org/articles/10.3389/fpsyt.2023.1231760/full#supplementary-material
