# Supplementary material for: Deep brain stimulation for substance use disorder: a systematic review and meta-analysis
Source: Front Psychiatry. 2023 Aug 10;14:1231760. doi: 10.3389/fpsyt.2023.1231760 (PMC10449586; doi:10.3389/fpsyt.2023.1231760)
Supplement: Supplementary file 1 [file Data_Sheet_1.docx]

***Supplementary***

**Search strategy**

**1-PubMed=199**

("Addiction Medicine"[Mesh] OR "Substance-Related Disorders"[Mesh] OR "Opioid-Related Disorders"[Mesh] OR "Cocaine-Related Disorders"[Mesh] OR "Amphetamine-Related Disorders"[Mesh] OR "Tobacco Use Disorder"[Mesh] OR "Morphine Dependence"[Mesh] OR "Heroin Dependence"[Mesh] OR "Alcoholism"[Mesh] OR "Narcotic-Related Disorders"[Mesh] OR "Opium Dependence"[Mesh])AND (("Deep Brain Stimulation"[Mesh]) OR ( “DBS”[Mesh]) )

**2-Scopus=321**

TITLE-ABS-KEY (( "Addiction Medicine" OR "Substance-Related Disorders" OR "Opioid-Related Disorders" OR "Cocaine-Related Disorders" OR "Amphetamine-Related Disorders" OR "Tobacco Use Disorder" OR "Morphine Dependence" OR "Heroin Dependence" OR "Alcoholism" OR "Narcotic-Related Disorders" OR "Opium Dependence" ) AND ( ( "Deep Brain Stimulation" ) OR ( "DBS" ) ) )

Web of Science Core Collection=77

("Addiction Medicine" OR "Substance-Related Disorders" OR "Opioid-Related Disorders" OR "Cocaine-Related Disorders" OR "Amphetamine-Related Disorders" OR "Tobacco Use Disorder" OR "Morphine Dependence" OR "Heroin Dependence" OR "Alcoholism" OR "Narcotic-Related Disorders" OR “Opium Dependence")AND (("Deep Brain Stimulation") OR OR ( “DBS”) )

**3-OVID=118**

("Addiction Medicine" OR "Substance-Related Disorders" OR "Opioid-Related Disorders" OR "Cocaine-Related Disorders" OR "Amphetamine-Related Disorders" OR "Tobacco Use Disorder" OR "Morphine Dependence" OR "Heroin Dependence" OR "Alcoholism" OR "Narcotic-Related Disorders" OR "Opium Dependence”) AND (("Deep Brain StimulationOR (“DBS”) ) {Including Related Terms}

| **Study** | **Year** | **Surgery Procedure** | **Electrode Model** | **Imaging Technique** | **Target Area** | **Stimulation Parameters** |
| --- | --- | --- | --- | --- | --- | --- |
| **Bach P** | **2023** | **Stimulation was started with cathodic monopolar stimulation.** | **-** | **-** | **NAc** | **Stimulation: 130 Hz, 90 µs, and 3.5 V** |
| **Davidson B** | **2022** | **DBS surgery was performed in a single stage.** | **Model 3387 (Medtronic)** | **Preoperative T1/T2 sequences obtained on a 3-tesla MRI** | **NAc** | **-** |
| **Mahoney JJ** | **2021** | **Bilateral quadripolar 3 mm electrodes were implanted.** | **Model 3387 (Medtronic)** | **-** | **-** | **Electrodes: 1−, 9−, 2+, 10+; Frequency: 145 Hz; Pulse Width: 90 μs; Intensity: 6 V** |
| **Zhu R** | **2020** | **Bilateral anterior capsulotomy and bilateral NAc DBS surgery were performed.** | **1210–40 (SceneRay)** | **High-resolution MRI and CT imaging** | **NAc and anterior capsulotomy** | **-** |
| **Chen L** | **2019** | **Surgery: All patients were implanted with bilateral DBS leads under general anesthesia with a trajectory through the ALIC into NAc.** | **Custom quadripolar electrodes (1.27 mm in diameter with four 3-mm stimulating contacts at intervals of 2.0, 4.0, and 4.0 mm)** | **MRI Scan: 3.0T MRI Scan (General Electric Company, United States)** | **NAc: Posterior portion** | **Parameters not provided** |
| **Müller UJ** | **2016** | **-** | **-** | **-** | **-** | **-** |
| **Gonçalves-Ferreira A** | **2016** | **Surgery: Bilateral electrodes (3387 electrodes connected to an Activa PC generator [Medtronic, Minneapolis]) were implanted under local anesthesia.** | **3387 electrodes** | **Neuronavigation (StealthStation; Medtronic) on fused magnetic resonance imaging and computed tomography images** | **Anterior limb of the internal capsule** | **Contacts 0 targeted the posterior Acc 3 mm below and behind the AC, contacts 1 were in the anterior-lateral edge of the BNST at the AC level, and contacts 2 and 3 were in the anterior limb of the internal capsule 3 mm and 6 mm above the AC. Stimulation parameters: Frequency: 130 Hz, Pulse width: 150 µsec, Voltage: 1–6 volts for initial parameters and active contacts bilaterally 0–1; right hemisphere: Frequency: 150 Hz, Pulse width: 150 µsec, Voltage: 3–4 volts; left hemisphere: Frequency: 150 Hz, Pulse width: 150 µsec, Voltage: 2.5–3 volts for definitive parameters.** |
| **Voges J** | **2013** | **DBS electrodes were implanted using a modified Riechert-Mundinger stereotactic frame.** | **Model 3387 (Medtronic)** | **Coronal MRI scans** | **NAc** | **-** |
| **Zhou H** | **2011** | **Surgery: Bilateral quadripolar DBS leads (Model 3387, Medtronic, Minneapolis, Minnesota) were implanted stereotactically into the bilateral NAc.** | **Model 3387** | **Not provided** | **Bilateral NAc** | **Stimulation parameters: Stimulation amplitude gradually increased from 0.8 V to 2.5** |
| **Kuhn J** | **2011** | **Bilateral stereotactically guided implantation of quadripolar electrodes was carried out.** | **Model 3387 (Medtronic)** | **Stereotactical X-ray** | **NAc** | **Stimulation: -0, -1, and +case (5.5 V, 120 µsec, 130 Hz)** |
| **Mantione M** | **2010** | **Surgery: Bilateral electrodes (Model 3389; Medtronics, Inc., Minneapolis, MN) were implanted in the nucleus accumbens under local anesthesia.** | **Model 3389** | **Computed tomography fused with preoperative magnetic resonance imaging** | **Nucleus accumbens** | **Stimulation parameters: Monopolar stimulation with contacts 0 and 1 (negative), Pulse width: 90 microseconds, Frequency: 185 Hz, Voltage: 3.5 V initially, then changed to contacts 2 and 3 (negative) after three weeks.** |
| **Kuhn J** | **2009** | **Quadripolar electrodes were implanted stereotactically in the NAc and the neighboring internal capsule.** | **Model 3387 (Medtronic)** | **CT and conventional X-ray procedures** | **NAc and anterior limb of internal capsule** | **-** |

**Supplementary; Table 1 presents information on surgery procedures, electrode models, imaging techniques, target areas, and stimulation parameters**
